# Epigenetic Inactivation of EFEMP1 Is Associated with Tumor Suppressive Function in Endometrial Carcinoma

**DOI:** 10.1371/journal.pone.0067458

**Published:** 2013-06-28

**Authors:** Tingting Yang, Haifeng Qiu, Wei Bao, Bilan Li, Cong Lu, Guiqiang Du, Xin Luo, Lihua Wang, Xiaoping Wan

**Affiliations:** 1 Department of Obstetrics and Gynecology, Shanghai First People’s Hospital Affiliated to Shanghai Jiao Tong University School of Medicine, Shanghai, China; 2 Department of Obstetrics and Gynecology, International Peace Maternity & Child Health Hospital Affiliated to Shanghai Jiao Tong University School of Medicine, Shanghai, China; 3 Department of the Center of Research Laboratory, International Peace Maternity & Child Health Hospital Affiliated to Shanghai Jiao Tong University School of Medicine, Shanghai, China; University of Texas Health Science Center, United States of America

## Abstract

**Objective:**

EFEMP1, the epidermal growth factor–containing fibulin-like extracellular matrix protein 1, functions as an oncogene or a tumor suppressor depending on the cancer types. In this study, we aim to determine whether EFEMP1 affects the tumorigenesis and progression of endometrial carcinoma.

**Methods:**

The expression of EFEMP1 was investigated using immunohistochemistry in a panel of normal endometrium (n = 40), atypical hyperplasia (n = 10) and endometrial carcinoma tissues (n = 84). Methylation status of the EFEMP1 promoter was detected by methylation-specific PCR (MSP) and bisulphite genomic sequencing. Up- or down-regulation of EFEMP1 were achieved by stable or transient transfection with pCMV6/GFP/Neo-EFEMP1 or pGPU6/GFP/Neo-shEFEMP1 respectively. Effects of EFEMP1 on tumor proliferation, invasion and migration were evaluated by MTT, plate colony formation, Transwell and wound healing assay. The nude mouse tumor xenograft assay was used to investigate function of EFEMP1 *in vivo*.

**Results:**

Compared with normal endometrium (32/40) and atypical hyperplasia (7/10), EFEMP1 expression was much lower in endometrial carcinoma tissues (16/84) (P<0.001 and P = 0.02). EFEMP1 promoter was hypermethylated in endometrial carcinoma tissues (67%) as compared to normal tissue (10%) and down-regulation of EFEMP1 was associated with promoter hypermethylation. Treatment with 5-aza-2′-deoxycytidine (5-aza-dC) and/or trichostatin A (TSA) altered EFEMP1 methylation status, and restored EFEMP1 expression. Moreover, EFEMP1 decreased secretion of MMPs and inhibited tumor cell proliferation, metastasis and invasion *in vitro* and suppressed tumorigenesis in nude mice. Besides, EFEMP1 increased expression of E-cadherin and suppressed expression of vimentin in endometrial carcinoma.

**Conclusion:**

EFEMP1 is a new candidate tumor suppressor gene in endometrial carcinoma, and is frequently silenced by promoter hypermethylation. It could inhibit tumor growth and invasion both *in vitro* and *in vivo.* Our findings propose that targeting EFEMP1 might offer future clinical utility in endometrial carcinoma.

## Introduction

Endometrial carcinoma (EC) is one of the most common cancers of the female reproductive system. In the United States, approximately 49,560 new cases of EC will be diagnosed in 2013 and 8,190 deaths are expected [1]. In China, the incidence of EC has increased abruptly and the age of patient at onset has also decreased due in part to many factors like obesity and lifestyle changes [2], [3]. From a clinical viewpoint, EC is divided into two major sub-types, which is a commonly applied basis to determine the risk of prognosis and therapy [4]. However, the value of this classification is limited, as more than 20% of type I cancers recur, and with a poor prognosis. By contrast, almost half of type II tumors do not recur and display a better result. This observation reflects, at least to some extend an incomplete understanding of the molecular genetics of endometrial carcinogenesis [5].

Over the past 15 years intensely studied, epigenetic mechanisms have been shown to play a key role in tumorigenesis and cancer progression. DNA methylation changes are the most commonly studied epigenetic alterations, which are heritable in the short term, induced by DNA methyltransferases (DNMTs) and do not involve mutations of the DNA structure itself [6]. In mammals, DNA methylation always occurs within CpG islands, which are present in approximately 70% of all mammalian promoters [7]. Prior work has shown that hypermethylation of multiple suppressor genes are implicated in gene silencing, and ultimately culminate in cancer [8], [9]. In addition, previous studies of endometrial carcinoma suggest that DNA methylation is a promising biomarker for cancer detection, and numerous genes inactivated by promoter hypermethylation including PTEN, 14-3-3σ, MLH1, HOXA11, E-cadherin, PAR4, among others have been found in endometrial carcinoma [10], [11], [12], [13].

Epidermal growth factor–containing fibulin-like extracellular matrix protein 1 (EFEMP1), which is also known as Fibulin-3, is a member of the fibulin family proteins, which are characterized by a tandem array of epidermal growth factor–like repeats and the fibulin-type COOH-terminal module [14]. Fibulins are widely distributed in the vasculature and elastic tissues, whose main function are to mediate homotypic cell-to-cell and heterotopic cell-to-matrix communication [15]. In addition EFEMP1 takes part in processes such as regulation of body weight and behavioral control [16].

In cancer, members of Fibulin 1, 2, 4, and 5 have been reported to play crucial roles in tumorigenesis [17]. However, the relationship between EFEMP1 and tumorigenesis remains some controversy. It has been previously shown that in human pancreatic adenocarcinoma, EFEMP1 expression promoted tumor growth both *in vitro* and *in vivo* [18]. Besides, in cervical cancer up-regulation of EFEMP1 not only promoted angiogenesis, but also was associated with lymph node metastasis [19]. By contrast, in sporadic breast cancer, it was reported that EFEMP1 expression decreased, which was associated with a worse prognosis [20]. These paradoxical effects of EFEMP1 in tumorigenesis prompted us to investigate the role of EFEMP1 in endometrial carcinoma.

Thus, the purpose of this study was to determine the relationship between the expression of EFEMP1 and endometrial carcinoma, and to explore whether DNA hypermethylation was the mechanism responsible for the dampened expression of EFEMP1. The regulatory roles of EFEMP1 were investigated both *in vitro* and *in vivo*. Moreover, the association of EFEMP1 expression with the epithelial-mesenchymal transition was preliminarily discussed in this report.

## Materials and Methods

### Ethics Statement

The Human Investigation Ethical Committee of the International Peace Maternity and Child Hospital Affiliated Shanghai Jiao Tong University approved this study. The samples of endometrial carcinoma and normal endometrial tissues were collected after written informed consent from the patients. Animal research was carried out in strict accordance with the recommendations in the Guideline for the Care and Use of Laboratory Animals of China. The protocol was approved by the Committee on the Ethics of Animal Experiments of the Obstetrical and Gynecological Hospital affiliated Fudan University (Permit Number: SYXK (hu) 2008–0064). All efforts were made to minimize suffering.

### Cell Culture and Treatment

Human endometrial carcinoma cell-lines including HEC-1B, RL95-2, ISK, SPEC2, AN3CA and KLE were obtained from the Chinese Academy of Sciences Committee Type Culture Collection (Shanghai, China). Cells were maintained at 37°C and in a fully humidified atmosphere containing 5% CO_2_ and cultured in Dulbecco’s modified Eagle’s medium (DMEM)/F12 (11030; Gibco, Auckland, NZ) supplemented with 10% fetal bovine serum (FBS) (16000-44; Gibco, Carlsbad, CA), 100 u/ml penicillin/streptomycin. For demethylation studies, cells were treated with 10 µM 5-aza-Dc and/or 200 ng/ml TSA (Sigma, St. Louis, MO, USA) for 96 h. For anchorage-independent culture, cells were seeded into six-well plates that were precoated with hydrogel (Sigma, St. Louis, MO, USA) and cultured for 4–5 days in DMEM/F12 supplemented with 10% FBS. We hypothesized that cell spheroids (cell-sph) with anchorage-independent culture following trypsinization maintained partial imitation of EC in the metastatic process [21].

### Transient and Stable Transfection

The plasmids pCMV6/GFP/Neo-EFEMP1 (Product code: POSE144005929, Genechem, Shanghai, China) containing transfection-ready EFEMP1 cDNA (GenBank: BC014410) were transfected into HEC-1B cells by Lipofectamine™ 2000 (Invitrogen, life technologies, USA) according to the manufacturer’s protocol. The cell-line HEC-1B-exEFEMP1 was established by maintaining selection pressure with G418 (1 mg/ml, Gibco, Carlsbad, CA, USA) in the growth medium and selection of resistant clones. Plasmids of shEFEMP1 (pGPU6/GFP/Neo-shEFEMP1, Shanghai Genepharma Ltd, China) (CACAACGUGUGCCAAGACAUA) were transfected into RL95-2 cells by Lipofectamine™ 2000. Selection pressure was maintained by 800 µg/ml of G418 (Gibco) in the culture medium. KLE was transiently transfected with shEFEMP1 in the absence of selection pressure. For negative controls, HEC-1B cells were transfected with a pure pCMV6/GFP/Neo vector and RL95-2 or KLE cells were transfected with the pGPU6/GFP/Neo vector respectively under the same culture conditions by Lipofectamin™ 2000. EFEMP1 expression of the transfected cell-lines were verified by qRT- PCR and Western immunoblotting.

### Tissue Collection

We obtained 84 cases of uterine endometrial carcinoma wax blocks and 97 cases of fresh endometrial carcinoma tissues that had been previously frozen at −80°C from patients who underwent initial hysterectomy from April 2007 through December 2011. The stages and histological grades of these tumors were established according to the criteria of FIGO (2009) (http://www.figo.org/). In addition 40 cases of normal endometrium and 10 cases of atypical hyperplastic samples were also obtained from patients who underwent hysterectomy to treat uterine myoma. An independent pathologist verified the histological diagnosis of all collected tissues.

### Immunohistochemistry

Tissue blocks were cut into 4 µm sections, which were incubated with rabbit polyclonal anti-EFEMP1 (diluted 1∶200; ab1426; ABCAM, Hong Kong), rabbit polyclonal anti-E-cadherin (diluted 1∶50; CST, USA), rabbit polyclonal anti-vimentin (diluted 1∶50; CST, USA) and Ki67 (diluted 1∶100; Epitomics, Burlingame, CA, USA) at 4°C overnight in humid champers. Two independent pathologists, who were blinded to the clinical and pathological data, evaluated the specimens. Sections were evaluated according to semi-quantitative immunoreactivity scores (IRS). Accordingly, the percentage of positive staining was graded as a PP-value (0 = negative, 1 = <25%, 2 = 25–50%, 3 = 50–75% and 4 = >75%) and a staining intensity score (SI-value: where 0 = none, 1 = weak, 2 = moderate, and 3 = strong). For each specimen, the value of the IRS was obtained by multiplying the PP and the SI scores [22]. We defined negative EFEMP1 expression if IRS<5 [23].

### Quantitative RT-PCR Analysis

Total RNA was extracted from cultured cells by Trizol Reagent (Invitrogen; life technologies, USA). The isolated RNA was reverse transcribed into complementary DNA with the one-step Prime Script RT reagent Kit (TaKaRa; Dalian, China), and the cDNA was analyzed by real-time PCR using SYBR Premix Ex Taq (TaKaRa) in an Eppendorf Mastercycler® realplex. The primers for this PCR were: sense5′-CAGGACACCGAAGAAACCAT -3′ and antisense 5′-GTTTCCTGCTGAGGCTTTC -3′, which produced an amplicon of 110 bp. Data analysis employed the 2(-ΔCt) method. Data were obtained in triplicate from three independent experiments.

### Bisulfite Modification and Bisulfite Genomic Sequencing

Genomic DNA was isolated using the DNA Extraction Mini Kit (TIANGEN Biotech, Beijing, China). Bisulfite modification was performed with the EZ DNA Methylation Gold Kit (ZYMO Research, Los Angeles, CA, USA) according to the manufacture’s instruction. Bisulfite-sequencing PCR for EFEMP1 was done using ZymoTaq™ PreMix (ZYMO Research). The primer pair for sequence was in the EFEMP1 promoter and exon 1 region (Figure S1), sense: 5′ -GGA GTT GGT AAG GGA GGG TAA-3′, and antisense: 5′-CAA CTC ACC CCA CCT CAC TC-3′, which amplify a 376 bp product. The PCR conditions were: denaturation at 95°C for 10 min, 40 cycles at 95°C for 30s, 58°C for 30s, and 72°C for 30s. PCR products were purified directly using the TIAN gel Midi Purification Kit (TIANGEN Biotech, Beijing, China) and ligated into the pGEM-T easy vector (Promega Corporation, Madison, WI, USA).

### Methylation-Specific PCR (MSP)

Methylation-specific PCR was carried out in a volume of 20 µl using ZymoTaq™ PreMix (ZYMO Research) containing 100 ng of bisulfite modified DNA. The PCR conditions were: 40 cycles at 95°C for 30s, 60°C for 30s, 72°C for 30s after 95°C for 10 min, and finally 72°C for 7 min. Primer pairs for the methylated and unmethylated regions of the EFEMP1 promoter were:

MF5′-CGTCGGGTTCGTAACGTTGG-3′, and MR5′-GACAACGACCGCGACGACAA-3′;

UF5′-GGTGTGGGGTTTTTGTGAGTTTG-3′, and UR5′-ACAACAAAATCCACACCCCCTA-3′.

The amplified DNA was analyzed on 2% agarose gels by standard electrophoresis.

### Western Immunoblotting

Total protein was extracted with lysis buffer (Beyotime, China) containing a 1% dilution of the protease inhibitor PMSF (Beyotime). Protein concentrations were determined by the BCA Protein Quantitative Analysis Kit (Thermo, USA). Equal proteins were loaded into each lane of a 10% SDS-PAGE gel for protein separation, and transferred to PVDF membranes (Millipore, USA). After blocking the membrane with 5% milk proteins for 2 h at room temperature, the membrane was incubated with primary antibodies against EFEMP1(1∶2000; ab1426; ABCAM, *HK*), E-cadherin (1∶2000,CST), vimentin (1∶1000,CST) and β-actin (1∶2000,CST) at 4°C overnight. The peroxidase-linked secondary anti-rabbit and anti-mouse antibodies were used to detect the bound primary antibodies. The relative expression of target proteins was described as a ratio relative to the expression of β-actin.

### Proliferation Assay

Cells and transfected cells were seeded into 96-well plates at 2.5×10^4^ cells/ml and cultured for 1–5 d at 37°C in an air/5% CO_2_ atmosphere. During the final 4 hours of culture, MTT reagent (Sigma; St. Louis, MO, USA) was added to the culture medium. Absorbance values were then measured at 490 nm with microplate reader (Bio-Rad model 680, USA). For the plate colony formation assay, 200 cells/well were seeded into 6-well plates. When clearly identifiable cell clones had formed, the cells were fixed and stained with crystal violet and observed under a light microscope. In each experiment and under each condition, proliferation was assessed in triplicate, and experiments were repeated at least twice.

### In vitro Cell Invasion and Migration Assay

Cell invasion assays were done using 6.5 mm transwell chambers equipped with 8.0 µm pore-size polycarbonate membranes. In these assays, the upper champers were first coated with 40 µl of Matrigel at a 1∶3 dilution (BD Biosciences), and incubated at 37°C for 2 h. Next, cells were suspended in serum-free medium and loaded onto the top chamber of the transwell insert at a density of 5×10^5^ to 1×10^6^ cells/ml (200 µl/chamber). The lower champers were filled with 500 µl DMEM/F12 supplemented with 10% FBS. After incubation at 37°C in an air/5% CO_2_ atmosphere for 48 h, non-invasive cells were physically scraped from the membrane with the cotton swabs. The cells that were attached to the underside of the membranes were fixed in 4% paraformaldehyde and stained with crystal violet. To obtain a measured value of cell invasion, cells that had migrated to the lower surface of the membranes were counted in five randomly selected fields of view (x 200 magnification) and the mean value was scored for every field. Wound healing assays were used to demonstrate cellular migration. Representative images were captured at the time of wounding, 6 h, 12 h, and 24 h later. All experiments were repeated at least three times.

### ELISA for the Determination of MMP-2 and MMP-9

Non-transfected and transfected HEC-1-B or RL95-2 cells were seeded into 24-well plates at a density of 1×10^6^ cells/ml, and cultured as previously described. Each supernatant was centrifuged at 200 g and stored at –80°C until assayed. The concentrations of human MMP-2, and MMP-9 were determined by ELISA Kit (R&D Systems, Minneapolis, USA) according to the manufacturer’s instructions. Values were read using microplate reader (Bio-Rad model 680, USA). Experiments were done in triplicate and in three independent experiments.

### Nude Mouse Tumor Xenograft Assay

Ten 6-wk old female BALB/c mice were obtained from Shanghai Life Science Institute (Slac Laboratory Animal Co., Ltd, China). To establish a nude mouse model bearing EC, stable HEC-1B cells transfected with the EFEMP1 gene (HEC-1B-exEFEMP1), and vector alone (HEC-1B-NC) were used. All mice were randomly divided into two groups of 5 mice. The cells were injected subcutaneously into the flank of each mouse at a density of 1×10^7^cells. When the tumor had formed approximately 10 d after injection, the size of the tumor was measured every 4 d and the tumor volume was calculated as (Rmax)×(R^2^ min)/2. Mice were sacrificed 4 wk post-injection, following which tumors were carefully removed, and measurements taken of their weight and volume prior to further histological evaluation.

### Statistical Analysis

Statistical analyses were made using the Statistical Package for the Social Sciences (SPSS) software version 17.0 (Chicago, IL, USA). Data was represented as the mean with one standard deviation (SD). Measured data was assessed by unpaired Student’s T-test, or one-way ANOVA for multiple comparisons, and χ^2^ test for 2×2 tables was used to compare the categorical data. Differences were considered statistically significant with an alpha value of P<0.05.

## Results

### Down-Regulation of EFEMP1 in Endometrial Carcinoma

**Figure 1:**
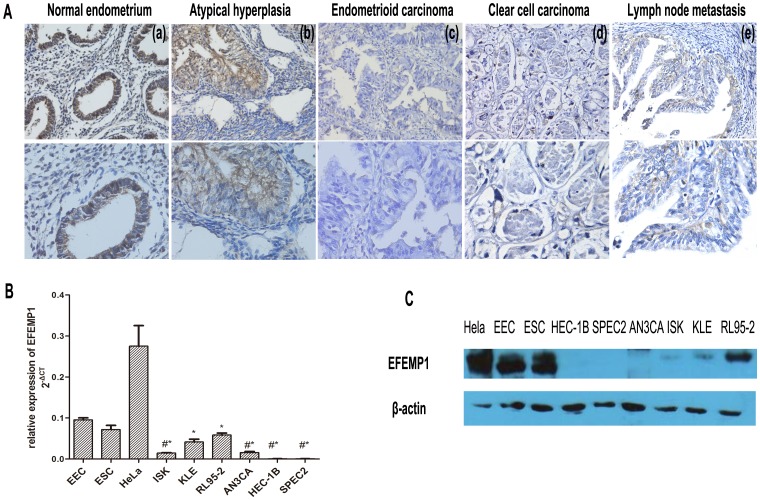
Differential EFEMP1 expression in normal endometrium and endometrial carcinoma. A: Immunochemistry of EFEMP1 in endometrial tissues. (a) normal endometrium with strong staining, (b) atypical hyperplasia with moderate staining, (c) endometrioid carcinoma with no staining, (d) clear cell carcinoma with no staining, and (e) serous endometrial carcinoma with lymph node metastasis. Magnification:×200 (above) and×400 (below). B: Down-regulation of EFEMP1 mRNA expression in EC cell-lines as compared with EEC (endometrial epithelial cell). ESC is the abbreviation of endometrial interstitial cell. HeLa cells were used as a positive control. The experiment was repeated twice, and each experiment was done in triplicate. * P<0.05, #*P<0.01. Arithmetic means of the data (bars) and SD (error bars) are shown. C: EFEMP1 expression in various endometrial cancer cell-lines as determined by Western immunoblotting analysis.

We used immunohistochemistry to compare the expression of EFEMP1 between normal endometrium, atypical hyperplasia and EC tissues. We observed positive expression of EFEMP1 in 19.1% (16/84) of carcinomas, which was significantly lower than that seen in 80% of normal endometrium (32/40) and 70% of atypical hyperplasia (7/10) respectively (P<0.001 and P = 0.02, Table 1, Table S1and S2). Representative differences in staining of EFEMP1 by normal and malignant endometrium are also shown (Figure 1A). Notably, EFEMP1 was largely localized to the cytoplasm and plasma membrane.

**Table 1:** Statistical difference between EFEMP1 expression in normal endometrium and endometrial carcinoma.

	EFEMP1 expression		?^2^	P*
	Negative	Positive		
Normal endometrium	8	32	40.166	<0.001
Endometrial carcinoma	68	16		
*χ^2^test				

To investigate whether the change in EFEMP1 expression of EC was associated with any of the available clinical characteristics, we studied the association of EFEMP1 expression levels with the clinical and pathological parameters of endometrial carcinoma (Table 2). Our observations showed that the positive expression of EFEMP1 was lower in stage I EC (8/62) as compared with either stage II (4/11) or stage III (4/11) tumors (P = 0.038). Expression of EFEMP1 was significantly decreased in patients showing lymph node metastasis (P = 0.014). However there were no significant differences among pathological grade, histological type, varying depth of myometrial invasion or in the context of involvement of the lymph vascular space.

**Table 2:** Immunohistochemical expression of EFEMP and its association with clinic-pathological variables.

Variable	No. patients (%)		EFEMP1 expression				
	n	%	Negative		Positive		P
Total	84	100					
Age (years)							0.731
≤50	40	47.6		33		7	
>50	44	52.4		35		9	
FIGO stage							0.038
Stage I	62	73.8		54		8	
Stage II	11	13.1		7		4	
Stage III	11	13.1		7		4	
Grade							0.374
G1	40	45.2		32		8	
G2	30	33.3		23		7	
G3	14	21.5		13		1	
Histological type							0.901
Endometrioid	70	83.3		56		14	
Nonendometriod	14	16.7		12		2	
Myometrial invasion							0.336
≤1/2	63	84.5		49		14	
>1/2	21	15.5		19		2	
Lymph node metastasis							0.014
No (N-)	57	91.7		42		15	
Yes(N+)	27	8.3		26		1	
Lymphovascula space involvement							0.597
No	56	66.7		46		10	
Yes	28	33.3		22		6	
ER expression							0.457
Negative	19	22.6		17		2	
Positive	65	77.4		51		14	
PR expression							0.563
Negative	18	21.4		16		2	
Positive	66	78.6		52		14	
p53 expression							0.529
Negative	66	78.6		53		13	
Positive	18	21.4		15		3	

In six EC cell-lines, EFEMP1 mRNA levels were significantly lower as compared with primary cultured endometrial epithelial cells (EEC), and HeLa cells acted as positive control (P<0.05, Figure 1B). Moreover, differential protein expression of EFEMP1 was consistent with results obtained for mRNA expression levels (Figure 1C).

### Promoter Hypermethylation in Endometrial Carcinoma

**Figure 2:**
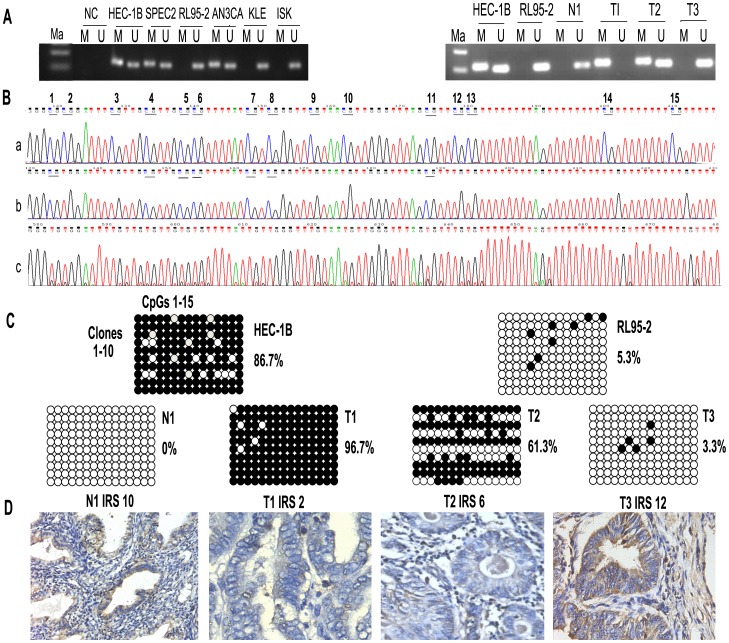
Methylation status of EFEMP1 in cell-lines and tissues. A: MSP was used to determine the methylation status of EFEMP1 in EC cells and tissues, NC = H_2_O; N = normal endometrial tissue; T = endometrial carcinoma; M = PCR products amplified by MSP specific for methylated DNA; U = PCR products amplified by MSP specific for unmethylated DNA. B: Showing three examples of the different methylation sequences as revealed by bisulfite genomic sequencing. (a) Shows a fully methylated sequence with 15 methylated CpGs. The cytosine nucleotides were not converted into thymines. (b)Shows a partly methylated sequence and only CpGs 1, 4, 5, 6, 7, 8 and 11 were methylated. (c) Shows a fully unmethylated sequence since all cytosine nucleotides of the 15 CpGs were converted into thymines. C: Bisulfite genomic sequencing detected endogenous levels of methylation among the endometrial carcinoma cell-lines HEC-1B and RL95-2, normal endometrium (N1) and endometrial carcinoma tissues (T1, T2, T3). Columns indicate CpG dinucleotides 1–15, and horizontal lines analyzed clones 1–10. Black dots symbolize methylated CpGs, and white dots symbolize unmethylated CpGs. D: Showing immunohistochemical staining of tissues with known methylation status as above. Magnification x400.

To explore the possible mechanisms, which may have contributed to the diminution in expression of EFEMP1, we used MSP and bisulfite genomic sequencing to examine the methylation status of the EFEMP1 promoter in a panel of EC cell-lines, primary endometrial carcinomas, and normal endometrial tissues. The results demonstrated that 67% (65/97) of EC tissues were methylated in the EFEMP1 promoter region, and only 10% (4/40) of normal endometrial tissues were methylated and typical images were shown in Figure 2A. Among six EC cell-lines, half of them including HEC-1B, SPEC2 and AN3CA displayed EFEMP1 promoter methylation (Figure 2A).

To confirm that the results of MSP were reliable, we performed direct sequencing analysis of a 376-bp fragment including 40 CpG dinucleotides in the promoter and exon 1 region. EFEMP1 gene was amplified with bisulfite-treated DNA, which was obtained from HEC-1B or RL95-2 cells and tissues of N1, T1, T2 and T3. However 25 CpG dinucleotides within and near exon1 were determined to be completely unmethylated, and differential methylation was observed in 15 CpG dinucleotides of the promoter (Figure 2B). We found that the percentage of methylated CpG dinucleotides in HEC-1B and RL95-2 was 86.7% and 5.3% respectively (Figure 2C).

By contrast, to further investigate whether methylation of the EFEMP1 promoter was associated with EFEMP1expression, tumor tissues displaying different methylation status were shown by immunohistochemical staining. In completely unmethylated normal endometrium (N1) and endometrial carcinoma (T3), the percentage of methylated CpG dinucleotides was 0% and 3.3% respectively, and showed strong expression of EFEMP1 (IRS10, 12). Tumor (T1) with the highest methylation status (96.7%) showed the lowest protein expression (IRS 2.0), whereas semi-methylated T2 with 61.3% methylation displayed moderate expression (IRS 6.0) (Figures 2C and 2D). In addition, 43 cases of EC tissues with the data of both MSP and immunohistochemistry were analyzed. We found that promoter methylation of EFEMP1 in endometrial carcinomas was associated with lower value of IRS (mean 1.8) as compared to carcinomas with unmethylated EFEMP1 (mean 4.080) (P = 0.0333, Figure S3). These founding therefore strongly supported the association of high methylation status with low levels of gene expression.

### Promoter Methylation Status and Expression of EFEMP1 after Treatment with 5-aza-dC and/or TSA

**Figure 3:**
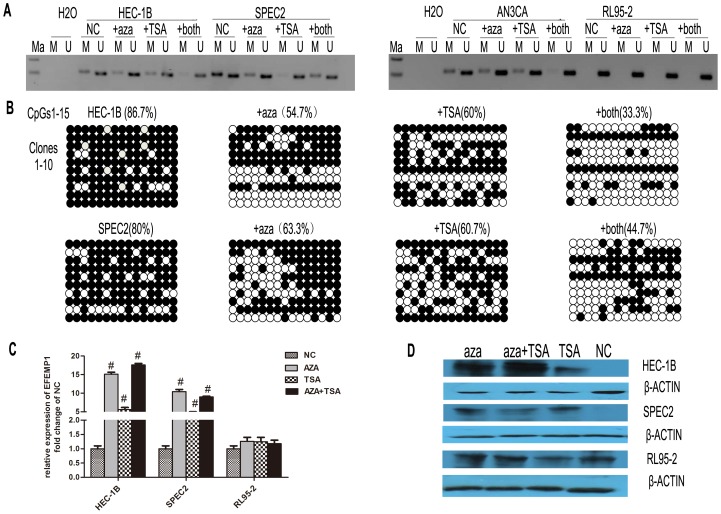
The effect of 5-aza-dC and TSA on EFEMP1 expression. A: Endometrial carcinoma cell-lines HEC-1B, AN3CA and SPEC2, shown to be methylated in the DNA promoter regain of EFEMP1, and RL95-2 shown to be unmethylated. Cells were treated with 5-aza-dC (10 µM) and/or TSA (200 ng/ml) for 96 h, and the methylation status of EFEMP1 was assessed by MSP. B: Bisulfite genomic sequencing detected endogenous levels of methylation before and after treatment with 5-aza-dC (10 µM) and/or TSA (200 ng/ml). Columns indicate clones 1–10; horizontal lines show the analysis of CpG dinucleotides 1–15. Black dots symbolize methylated CpGs, and white dots symbolize unmethylated CpGs. C: Showing qRT-PCR analysis and evaluation of EFEMP1 mRNA expression before and after treatment with 5-aza-dC and/or TSA. Data representative of three independent experiments are shown. # P<0.05. D: Western immunoblot showing changes in EFEMP1 protein expression. Protein samples were extracted from cells that were treated with 5-aza-dC and/or TSA.

To explore the epigenetic mechanism that participated in the regulation of EFEMP1 transcription, we utilized the following specific cell-lines: HEC-1B, SPEC2, AN3CA and RL95-2. We cultured HEC-1B, SPEC2, AN3CA and RL95-2 cells in the presence of 10 µM of 5-aza-dC or 200 ng/ml of TSA for 96 h. To study the effects of DNA methyltransferases inhibitor (DNMTi) and histone deacetylase inhibitor (HDACi) on DNA methylation of EFEMP1, we treated cells with 5-aza-dC for 48 h followed by TSA for an additional 48 h. The results of MSP analysis showed that both 5-aza-dC and TSA used alone or in combination altered EFEMP1 promoter methylation status in HEC-1B, AN3CA and SPEC2 cells (Figure 3A). Through analyzing the results of bisulfate genomic sequencing, we also observed that methylation status was partly reversed after treatment with 5-aza-dC and/or TSA (Figure 3B).

Moreover, we assessed the expression of EFEMP1 before and after treatment with 5-aza-dC with or without TSA co-treatment. After treatment with 5-aza-dC and/or TSA, we found that HEC-1B and SPEC2 cells showed reactivation of both EFEMP1 mRNA and protein expression (Figures 3C and 3D). In RL95-2 cells, in which no methylation was detected, we did not find any change in the expression of EFEMP1after treatment.

### EFEMP1 Inhibits Cell Growth *in vivo* and *in vitro*

**Figure 4:**
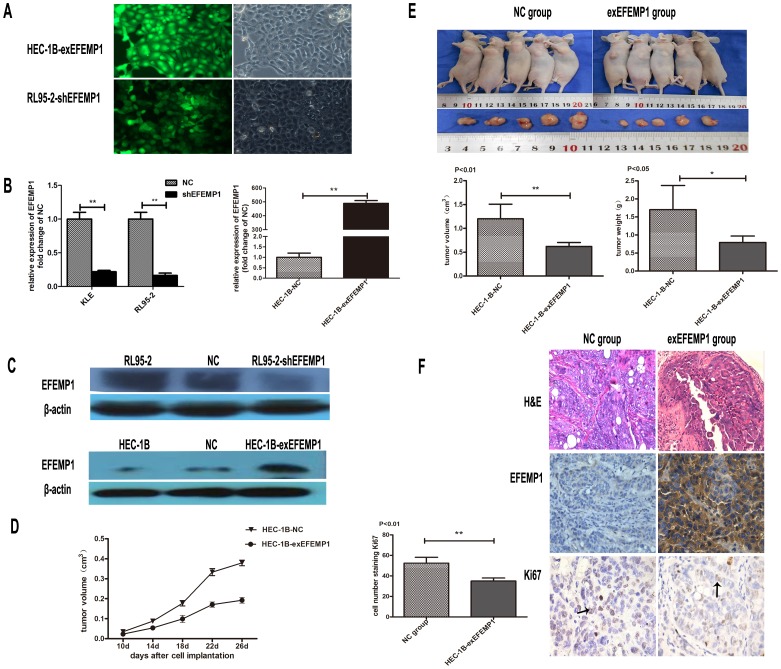
The effect of EFEMP1 on tumor growth *in vivo*. A: Stable transfection of HEC-1B cells and RL95-2 cells with pCMV6/GFP/Neo-EFEMP1 and shEFEMP1. The percentage of transfected cells with fluorescence was >95%. B: Levels of mRNA expression of EFEMP1 as evaluated by qRT-PCR after transfecting cells with these plasmids. The experiments here were repeated twice, and each was done in triplicate, ** P<0.01. C: Western immunoblot to demonstrate the efficacy of these plasmids. D: Showing the comparative growth rate of tumors formed from HEC-1B-NC and HEC-1B-exEFEMP1. Ten days after injection the size of the tumor was measured every 4 days. P<0.05. E: Four wks after injection of HEC-1B-NC and HEC-1B-exEFEMP1, the weight and volume of the tumors were measured. Arithmetic means (bars) and SD (error bars) are shown, * P<0.05, **P<0.01. F: Nude mice tumor tissues were paraffin embedded and tumor slides were stained with hematoxylin and eosin (magnification was ×400), antibody of EFEMP1 (× 400) and Ki67 (×400). The black arrow indicates positively-stained cells for Ki67, at least five regions were randomly selected to score positive cells (mean ± SD, P<0.01).

To perform cell function experiments, stable cell-lines with interference or over-expression of EFEMP1 were established (Figure 4A). The efficiency of shRNA effects was up to 80%. In addition, HEC-1B cells transfected with EFEMP1 gene was more than 500 times compared with cells transfected with pure vector (Figure 4B and 4C).

Nude mouse models of EC were successfully established using HEC-1B cells stably transfected with either the EFEMP1 gene (HEC-1B-exEFEMP1) or pure vector (HEC-1B-NC). We then determined tumor growth *in vivo* according to the tumor volume, which was measured every 4 day from 10 days after the injection. The observations indicated that tumors in the HEC-1B-exEFEMP1 group showed a lower growth rate than those tumors of the HEC-1B-NC group (P<0.05, Figure 4D). After the 4th week of injection, tumors were completely removed from the mice. We found that the final mean weight and volume of the HEC-1B-exEFEMP1 group was significantly lower as compared with the HEC-1B-NC group (P<0.05 and P<0.01, Figure 4E). Moreover, the tumors were sectioned and stained with hematoxylin and eosin, and immunohistochemically stained with antibodies against EFEMP1 or Ki67. We found a tight and nest-shaped distribution with occasional gland-like structures present. The staining of HEC-1B-exEFEMP1 tumors with Ki67 showed fewer instances of this marker as compared with the negative control group (P<0.01, Figure 4F).

**Figure 5:**
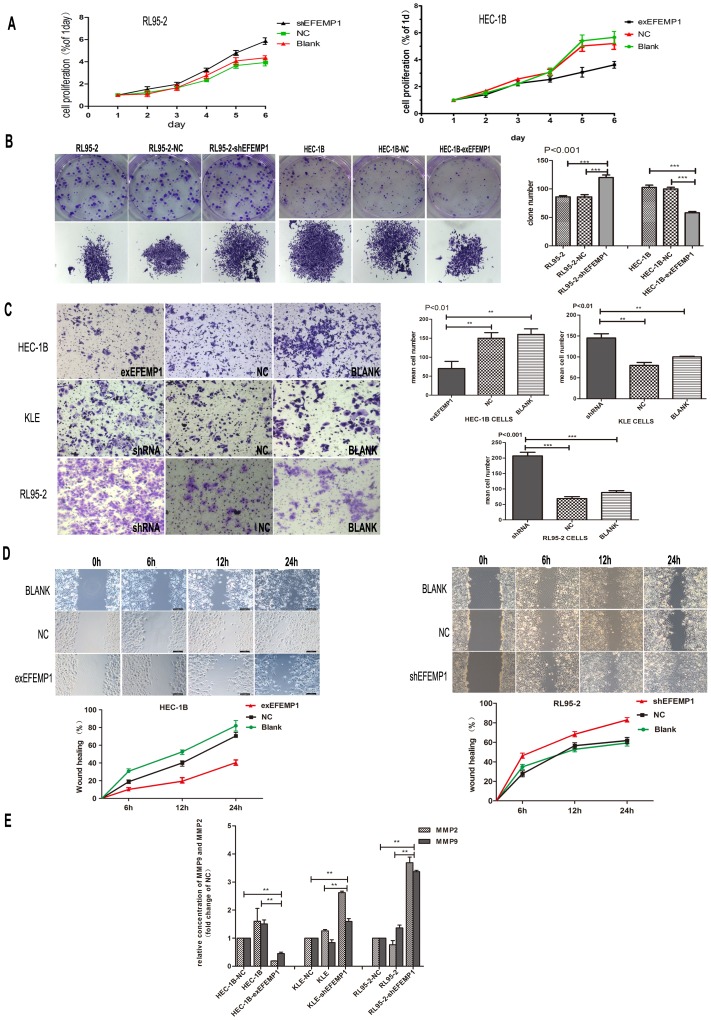
Effect of EFEMP1 on endometrial carcinoma cell proliferation, migration and invasion. A: Knockdown of EFEMP1 promoted cell growth in RL95-2. However cell proliferation was lower in HEC-1B-exEFEMP1 cells compared with negative control and HEC-1B. B: Plate colony formation assay was used to measure cell proliferation of EFEMP1 in HEC-1B-exEFEMP1 and RL95-2-shEFEMP1 and negative control. C: Representative images and statistical plots of Transwell assays in HEC-1B cells, which were stably transfected with exEFEMP1and vector control; RL95-2 cells and KLE cells transfected with shEFEMP1 and vector control (x200 magnification). The data were described as arithmetic mean ± SD of three independent experiments. D: Wound healing assays of cells expressing the vector control and exEFEMP1 in HEC-1B; and RL95-2 cells showing dampened expression of EFEMP1and vector control. Typical images were captured at the precise time of wounding at 0, 6, 12 and 24 h (original magnification×100). The percent of wound closure was measured in at least three randomly selected regions (mean ± SD). E: MMP-2 and MMP-9 secretion was decreased significantly in HEC-1B transfected with exEFEMP1 and increased in KLE and RL95-2 transfected with shEFEMP1cells. Data are expressed as mean concentrations ± SD of three independent experiments. * P<0.05, **P<0.01, ***P<0.001.

In vitro, the MTT proliferation assay revealed that down-regulation of EFEMP1 promoted cell growth in RL95-2 cells in a time-dependent manner. Additionally, over-expression of EFEMP1 inhibited cell growth in HEC-1B cells (Figure 5A). To further explore the role of EFEMP1 in cell growth, we studied plate colony formation assays. We found more pronounced and frequent colony formation in HEC-1B and HEC-1B-NC cells than was found in HEC-1B-exEFEMP1 cells (P<0.001). However in RL95-2-shEFEMP1 cells the ability to form colonies was more pronounced than RL95-2 and RL95-2-NC cells (P<0.001, Figure 5B, Figure S2).

### EFEMP1 Inhibited Tumor Migration and Invasion, and Decreased Secretion of MMPs in EC Cells

Immunohistochemistry revealed that patients with lymph node metastasis displayed weaker EFEMP1 expression. With this observation in mind, we hypothesized that functional expression of EFEMP1 may dampen tumor metastasis in EC. Thus, to test this hypothesis, we used Transwell assays with the following EC cell-lines HEC-1B, RL95-2 and KEL to model tumor invasion. We found that the invasive ability of HEC-1B-exEFEMP1 cells was approximately 2.5-fold lower than that shown in HEC-1B-NC and HEC-1B cells (P<0.01). However, after interfering with EFEMP1 expression in RL95-2 (RL95-2-shEFEMP1) and KLE (KLE-shEFEMP1) cells, the invasive ability was about 3-fold and 2-fold higher than both negative control and untreated cells (P<0.001 and P<0.01, Figure 5C). In wound healing assays, at 24 h after establishing the wound, HEC-1B-NC cells achieved almost complete wound closure (70.5%), whereas HEC-1B-exEFEMP1 was only 40.38%. We also found that wound closure in RL95-2-shEFEMP1 cells (81.5%) was much higher than that found in the negative control (60.2%) (P<0.001, Figure 5D).

To further explore the mechanism of EFEMP1 on tumor invasion and migration, we assayed culture supernatants from HEC-1B, RL95-2 and transfected cells for the concentration of MMP-2 and MMP-9. Over-expression of EFEMP1 was associated with reduced secretion of both MMP-2 and MMP-9, and decreased expression of EFEMP1 promoted secretion of both MMP-2 and MMP-9 (P<0.01, Figure 5E).

### EFEM1 Expression Influences both E-cadherin and Vimentin Expression in EC

**Figure 6:**
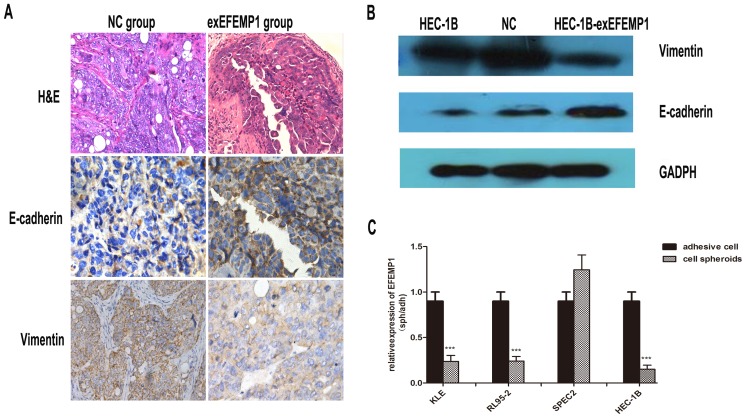
The effect of EFEMP1 on E-cadherin and vimentin expression. A: The nude mouse tumor tissues were paraffin embedded and tumor slides were stained with hematoxylin & eosin (×400), antibody of E-cadherin(× 400) and vimentin (×400). B: Western immunoblot analysis of the changes in expression of E-cadherin and vimentin in HEC-1B cells transfected with exEFEMP1 and vector. C: Cell spheroids cultured to mimic metastatic cancer cells. qRT-PCR analysis of the changes in EFEMP1 expression in various EC cells. Data are expressed as the mean; bars, ±SD, and normalized to adherent cells from three independent experiments. ***P<0.001.

In nude mice tumors, immunohistochemical analysis showed that the expression of E-cadherin in the HEC-1B-exEFEMP1 group was higher than that found in the control group. Additionally, the expression of vimentin was lower than that found in the control group (Figure 6A). We also found that HEC-1B cells transfected with EFEMP1 expressed higher levels of E-cadherin but lower levels of vimentin as compared with cells transfected with vector alone or untreated cells (Figure 6B). Moreover, to further elucidate the function of EFEMP1, we studied spheroids of endometrial cells in six-well culture plates coated with hydrogel to partially imitate the metastatic characteristics of EC. We found that cultured cellular spheroids expressed lower EFEMP1 mRNA levels as compared with cells cultured under normal culture plate conditions (Figure 6C).

## Discussion

In this study, we firstly reported that EFEMP1 was commonly lost in endometrial carcinoma, which was mainly caused by promoter hypermethylation. The suppressive effects of EFEMP1 on endometrial carcinoma were confirmed by both *in vitro* and *in vivo* studies, where cancer cell proliferation, invasion, and migration were found to be impaired. Moreover, EFEMP1 might have affected the process of EMT.

Immunohistochemical staining showed that EFEMP1 expression was significantly down- regulated in EC tissues and cell-lines as compared with normal endometrium and cells. However, the results that we obtained contradict some previously published work. For example, in cervical cancer [19] and pancreatic carcinoma [18], EFEMP1 expression was overexpressed and exerted an angiogenic effect during tumorigenesis. This phenomenon is not without precedent, since other tumor types have demonstrated context-specific expression of fibulin family members such as the expression of Fibulin-5 [24].

One of the major mechanisms that contribute to the process of tumorigenesis is the inactivation of tumor suppressor genes (TSGs). Inactivation of TSGs is always induced by various pathways including loss of function mutations in the coding site, chromosomal deletions, and epigenetic alterations in the DNA promoter regions rich in CpG islands or histone modification [25]. Most previous studies acknowledge that abnormal epigenetic alterations of TSGs (e.g., PTEN, 14-3-3σ, MLH1, HOXA11) contribute to endometrial carcinoma. Our observations showed in endometrial carcinoma, down-regulated EFEMP1 expression was largely caused by aberrant methylation and significantly suppressed cell proliferation, invasion and migration. This finding is consistent with other studies, which have shown lower expression of EFEMP1 in breast, lung and prostate cancer because of DNA promoter hypermethylation [20], [26], [27]. Our present results indicate that promoter hypermethylation is a major mechanism for inactivation of EFEMP1 as a tumor suppressor in endometrial carcinoma.

In clinic, EC can be diagnosed at an early stage of development because of its typical clinical manifestation, and then displays a promising prognosis. However, patients that present with tumor metastases invariably have a much worse prognosis [28]. At present, an effective and noninvasive method to forecast endometrial carcinoma at early stage and poor prognosis is lacking [29]. Recently, in pleural mesothelioma, it was found that EFEMP1 was a sensitive and specific biomarker that could distinguish healthy individuals with exposure to asbestos from patients with mesothelioma [30]. In our study, immunohistochemical data showed the low expression of EFEMP1 was associated with lymph node metastasis, and that cell function assays indicated the ability of EFEMP1 to dampen both the metastases and potential invasiveness of the primary tumor. This new function of EFEMP1 could serve as a predictive biomarker in endometrial carcinoma to predicate endometrial carcinoma.

In cancer cells, DNA methylation and histone deacetylation are known to contribute to gene silencing. In clinical and preclinical research some drugs of DNMTi and HDACi are able to reverse some of the aberrant gene repression and induce growth arrest, differentiation, and apoptosis in malignant cells [7]. Our study showed that both 5-aza-dC and TSA alone or in combination reversed promoter methylation status of EFEMP1, and significantly restored both transcription and protein expression of EFEMP1. This indicates that in EC cells 5-aza-dC and TSA are both associated with demethylation of EFEMP1. However it is conventionally accepted that expression of methylated genes can only be restored by DNA demethylated reagents, such as 5-aza-dC, and that inhibition of histone deacetylation by TSA only activates transcription of unmethylated genes [31].

One mechanism might be the role played by the methyl-binding proteins (MBDs) to CpG sequences. Several recently studies have indicated that MBDs including MBD1, MBD2, MBD3, and MeCP2 recruited histone deacetylase complexes to methylated DNA, which lead to histone deacetylation and chromatin condensation [32]. However, others reported that histone deacetylase recruited DNA methyltransferases to specific genes and thus targeted DNA methylation [33]. Recent study showed TSA was associated with MAGEA1 DNA demethylation in a variety of tumors [34]. These evidences show a reversible crosstalk between histone deacetylation and DNA methylation. Our observation that inhibition of histone deacetylation by TSA partially relieved transcriptional repression of the methylated EFEMP1 in endometrial carcinoma cells is consistent with this model. However the absolute mechanism of this synergistic effect remains unclear, and needs further investigation.

Several previous studies have identified key mediators of tumor metastasis including tumor extravasation, microenvironment remodeling, homing, invasion, migration, and survival, all of which have been shown to dramatically impact the peripheral metastatic ability of tumor cells [35]. Among many proteins present in the tumor microenvironment, E-cadherin and vimentin were shown to be important in cell-to-cell adhesion [36]. In our study, EFEMP1 down-regulated the expression of vimentin and increased E-cadherin expression levels *in vitro* and *in vivo*. Besides, EFEMP1 suppressed the secretion of MMP2 and MMP9. Furthermore EFEMP1 was found to strongly interact with other extracellular matrix (ECM) proteins such as TIMP3, and blocked the functional activity of VEGF, and inhibited tumor metastasis [37]. However, in endometrial carcinoma, further molecular studies are needed to elucidate the mechanism of EFEMP1 on suppression of tumor metastasis.

In addition, the expression of EFEMP also influences epithelial mesenchymal transition (EMT). During this process, endometrial epithelial cells acquire phenotypes of motile fibroblasts, which have been demonstrated in endometrial carcinoma [38]. In our study, to model cancer cell metastasis, we cultured anchorage-independent cells spheroids following trypsinization. The results demonstrated that EFEMP1expression was lower in cells cultured suspension as compared with cells cultured under plate conditions. Previous studies have shown that reasons contributing to EMT were various. For example, the cell signaling of integrin pathways, activation of TGF-β, and enhanced expression of tumor-associated MMPs [39]. Our observations just indicated that in EC, ectopic EFEMP1expression was associated with EMT, and most likely through disturbing ECM such as E-cadherin, vimtenin, MMP2 and MMP9. However this is not the only mechanism which requires further exploration.

In summary, our studies demonstrate the importance of EFEMP1 expression in preventing tumor proliferation and invasion in endometrial carcinoma and show that its expression is mainly regulated by promoter methylation. In particular, we have shown metastatic suppression of EFEMP1 and its association with EMT, which is the first mention in cancer. Future studies should elucidate the potential use of EFEMP1 methylation as a molecular biomarker to predict the risk of endometrial carcinoma development and its association with hormonal signaling such as estrogen. These studies would help guide the development and provision of new targeting therapies to treatment endometrial carcinoma patients.

## Supporting Information

Figure S1 **Diagram of EFEMP1 promoter and exon1 regain.** The primer sequences used in this study are underlined. “Star” symbolizes the transcription start site of. The “exon 1” symbolizes the transcriptional end of exon1.
(TIF)

Figure S2 **Statistical analyses of plate colony formation assay.** In plate colony formation assay, the mean cell numbers of single colone in HEC-1B, RL95-2 and transfected cells are counted in at least five randomly selected clones (mean ± SD). ***P<0.001.
(TIF)

Figure S3 **Relationship between EFEMP1 expression and methylation status.** This analysis was performed in 43 clinical samples having both IRS of immunohistochemistry and MSP data. IRS change is expressed as the difference between methylation and unmethylation endometrial carcinomas. In methylated EC, the mean value of IRS is 1.833, and in unmethylated EC the mean value of IRS is 4.080. P<0.05.
(TIF)

Table S1 Statistical difference EFEMP1 expression between aypical hyperplasia and endometrial carcinoma.
(DOCX)

Table S2 Statistical difference EFEMP1 expression between atypical hyperplasia and normal endometrium.
(DOCX)
