# Supplementary figures and images for: Epigenetic Inactivation of EFEMP1 Is Associated with Tumor Suppressive Function in Endometrial Carcinoma
Source: PLoS One. 2013 Jun 28;8(6):e67458. doi: 10.1371/journal.pone.0067458 (PMC3696089; doi:10.1371/journal.pone.0067458)

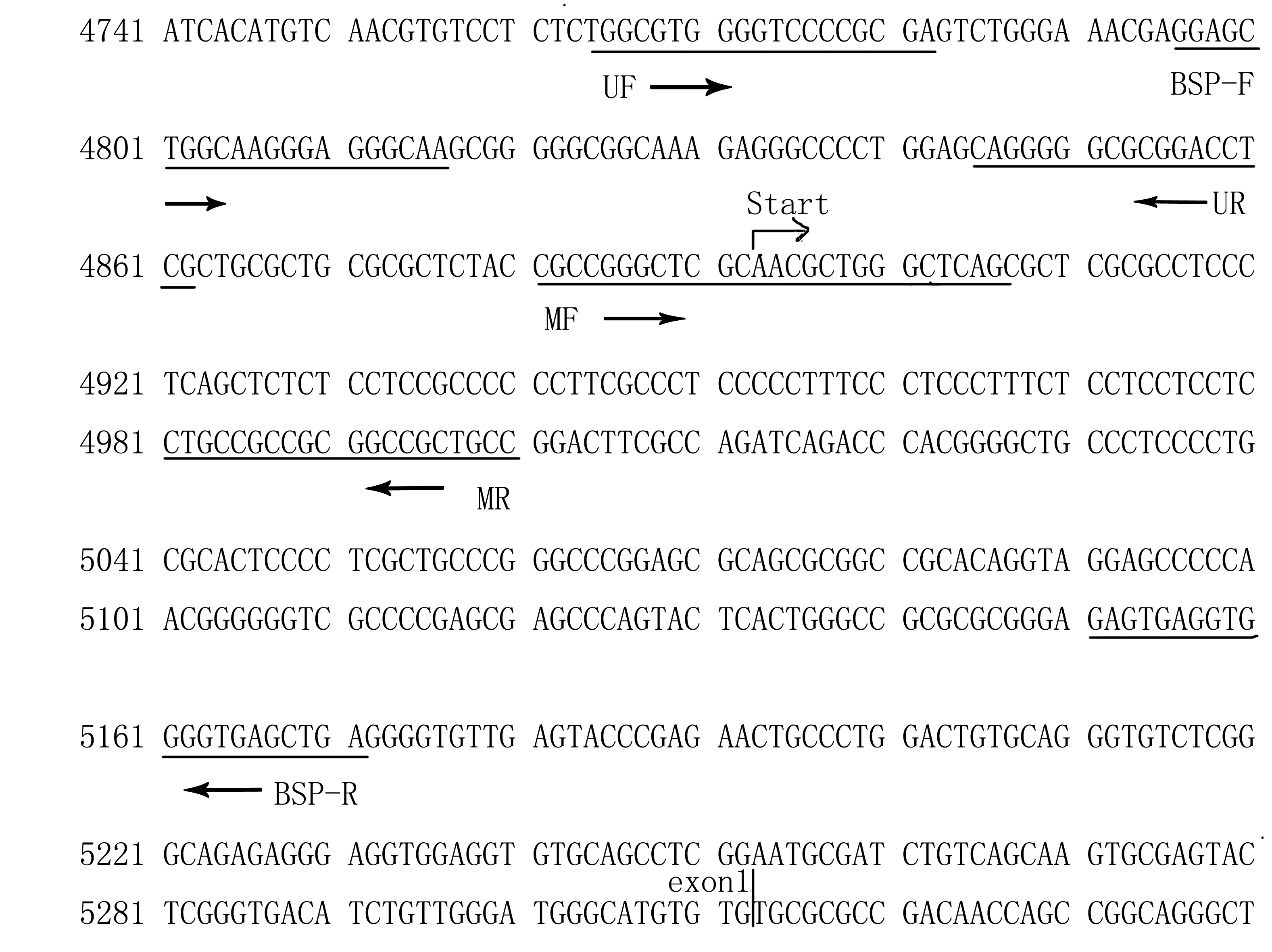

Supplement: Figure S1 — Diagram of EFEMP1 promoter and exon1 regain. The primer sequences used in this study are underlined. “Star” symbolizes the transcription start site of. The “exon 1” symbolizes the transcriptional end of exon1. (TIF) [file pone.0067458.s001.tif]

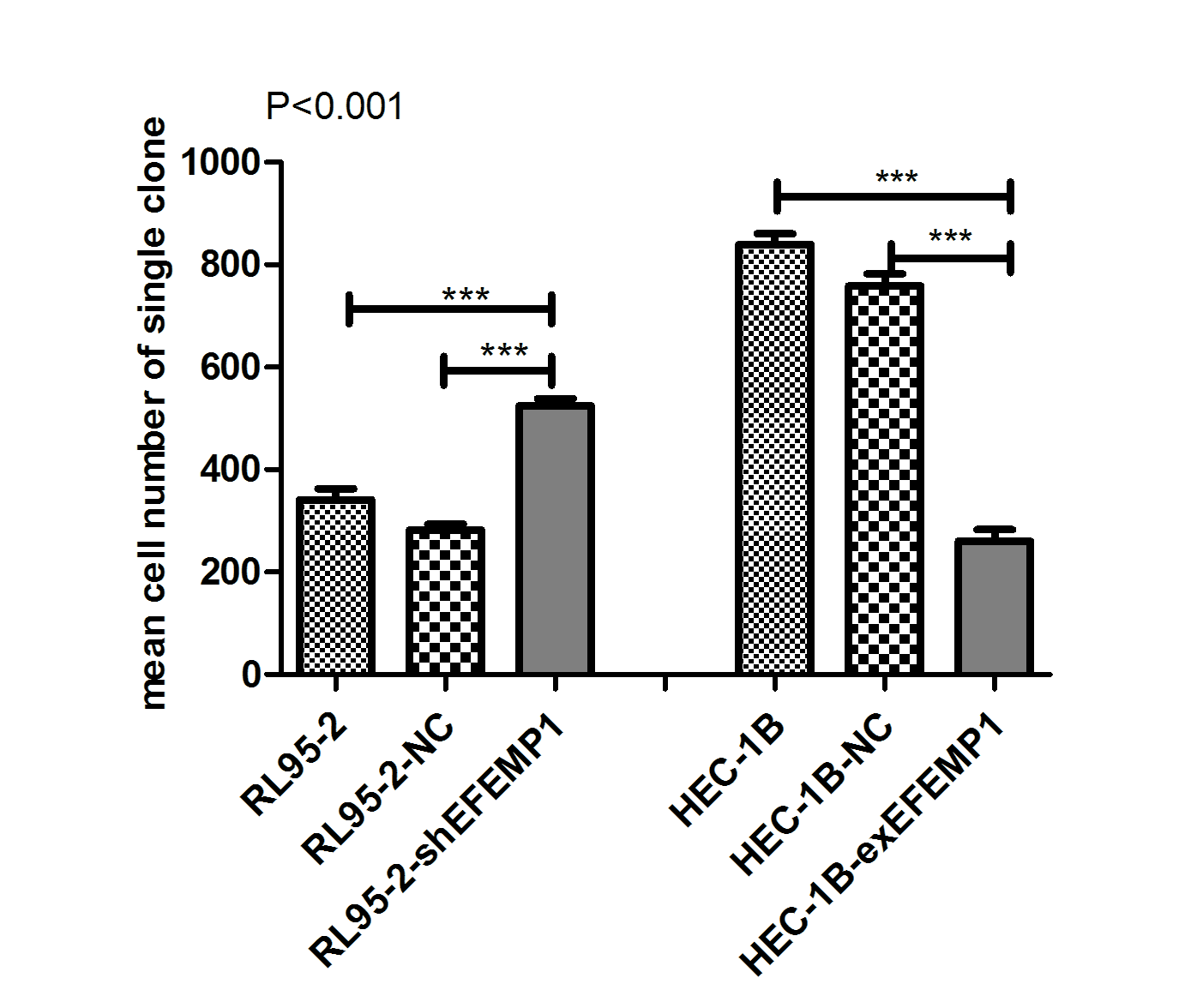

Supplement: Figure S2 — Statistical analyses of plate colony formation assay. In plate colony formation assay, the mean cell numbers of single colone in HEC-1B, RL95-2 and transfected cells are counted in at least five randomly selected clones (mean ± SD). ***P<0.001. (TIF) [file pone.0067458.s002.tif]

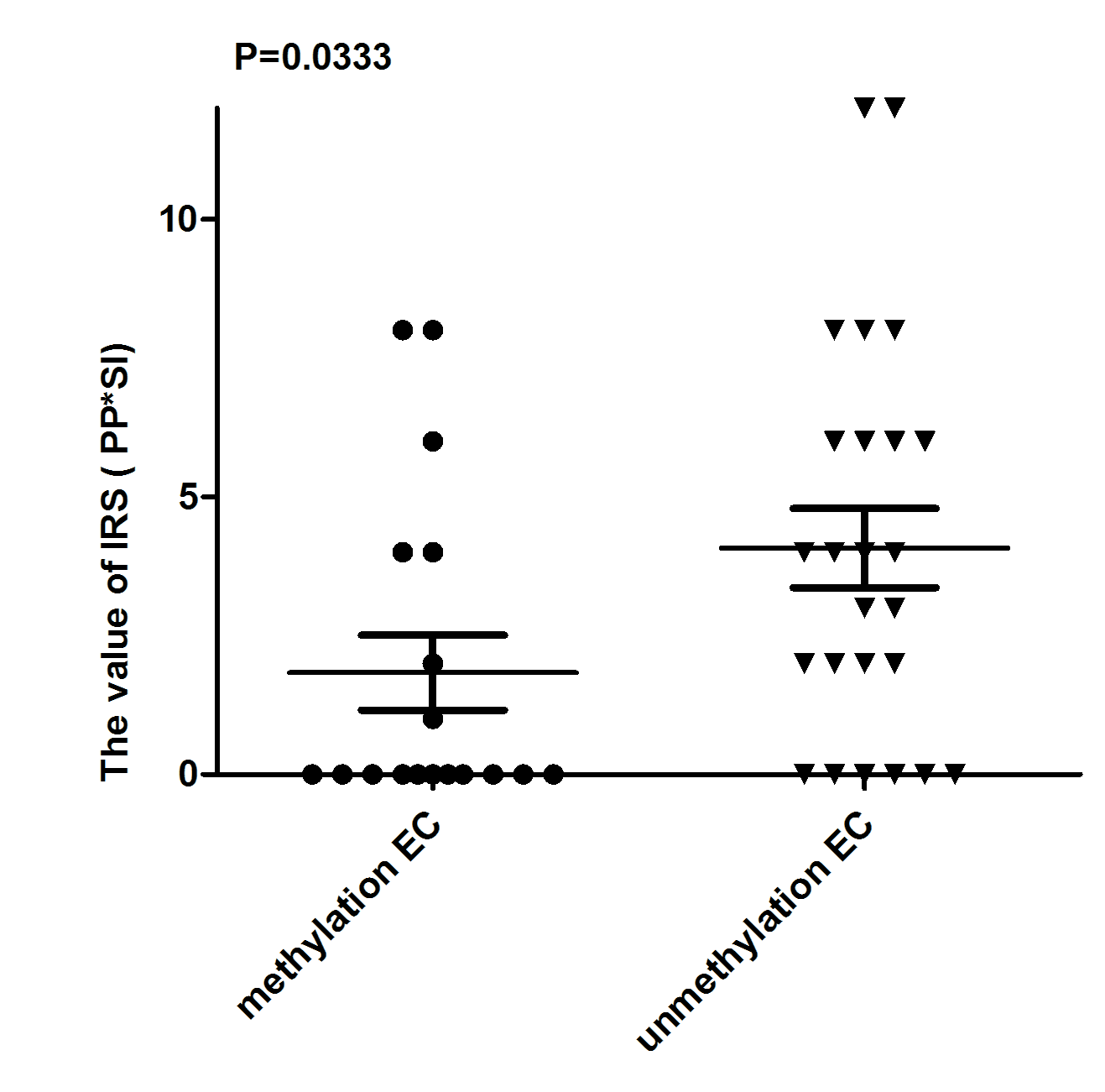

Supplement: Figure S3 — Relationship between EFEMP1 expression and methylation status. This analysis was performed in 43 clinical samples having both IRS of immunohistochemistry and MSP data. IRS change is expressed as the difference between methylation and unmethylation endometrial carcinomas. In methylated EC, the mean value of IRS is 1.833, and in unmethylated EC the mean value of IRS is 4.080. P<0.05. (TIF) [file pone.0067458.s003.tif]
