# Supplementary material for: Epigenetic Inactivation of EFEMP1 Is Associated with Tumor Suppressive Function in Endometrial Carcinoma
Source: PLoS One. 2013 Jun 28;8(6):e67458. doi: 10.1371/journal.pone.0067458 (PMC3696089; doi:10.1371/journal.pone.0067458)
Supplement: Table S1 — Statistical difference EFEMP1 expression between aypical hyperplasia and endometrial carcinoma. (DOCX) [file pone.0067458.s004.docx]

**Table S1. Statistical difference EFEMP1 expression between aypical hyperplasia and endometrial carcinoma**

|  | EFEMP1 expression | | **χ^2^** | P* |
| --- | --- | --- | --- | --- |
|  | Negative | Positive |  |  |
| Atypical hyperplasia | 3 | 7 | 9.947 | 0.02 |
| Endometrial carcinoma | 68 | 16 |  |  |
| ***χ^2^**test |  |  |  |  |
